# Immunohistochemical analysis reveals higher Myxovirus resistance protein 1 expression and increased macrophage count in placentas from patients with systemic rheumatic diseases

**DOI:** 10.1007/s00296-025-05856-w

**Published:** 2025-04-04

**Authors:** Juan J. Fierro, Mirthe H. Schoots, Silvia C. Liefers, Berber Doornbos-van der Meer, Gilles F. H. Diercks, Hendrika Bootsma, Jelmer R. Prins, Johanna Westra, Karina de Leeuw

**Affiliations:** 1 Department of Rheumatology and Clinical Immunology, University Medical Center Groningen, University of Groningen, Hanzeplein 1, Groningen, 9700RB The Netherlands; 2 Grupo Reproducción, Departamento de Microbiología y Parasitología, Universidad de Antioquia UdeA, Medellín, Colombia; 3 Department of Pathology and Medical Biology, University Medical Center Groningen, University of Groningen, Groningen, The Netherlands; 4 Department of Obstetrics and Gynecology, University Medical Center Groningen, University of Groningen, Groningen, The Netherlands

**Keywords:** Placenta, Macrophages, Systemic lupus erythematosus, Primary Sjögren’s disease, Antiphospholipid syndrome, Pregnancy, Interferons

## Abstract

To compare immune cell subsets and interferon (IFN) expression in placentas from patients with systemic lupus erythematosus (SLE), primary Sjögren’s disease (pSjD), antiphospholipid syndrome (APS), healthy controls (HC) and of women with adverse pregnancy outcomes (APO) without these systemic rheumatic diseases (SRD). Placenta biopsies from HC, SLE, pSjD, APS, and patients with fetal growth restriction (FGR), spontaneous preterm birth (PTB), or FGR and preeclampsia (FGR/PE) attended between 2008 and 2022 were recovered from the pathology biobank of the University Medical Center Groningen. Clinical characteristics and APO were retrieved from medical records. Immunohistochemistry was performed for Myxovirus resistance protein 1 (MxA), CD3, CD20, CD56, CD68, CD123, and Foxp3. The proportion of positive cells was established using an automated detection classifier, while MxA expression was assessed semi/quantitatively discriminating between maternal (decidua) and fetal (villi) tissue. Finally, placental lesion classification was performed. Our study included placentas from 11 SLE, 4 pSjD, 8 APS, 4 PTB, 8 FGR, 8 FGR/PE patients and 11 HC. A high rate of APO (70%) was identified in SRD patients. Patients with SRD had a higher macrophage (CD68+) count in decidua and villi than HC, but no differences were observed in T (CD3+), B (CD20+), NK (CD56+) and T regulatory (Foxp3+) cell count. No plasmacytoid dendritic cells (CD123+) were identified. Furthermore, patients with these SRD had higher MxA values than HC in villi but not in decidua. SLE, pSjD and APS patients have an increased macrophage count and interferon upregulation in the placenta compared to HC. Therefore, a pro-inflammatory environment might be key inducing placental dysfunction, which may lead to subsequent APO development.

**Supplementary Information:**

The online version contains supplementary material available at 10.1007/s00296-025-05856-w.

## Introduction

Systemic lupus erythematosus (SLE), primary Sjögren’s disease (pSjD) and antiphospholipid syndrome (APS) are systemic rheumatic diseases (SRD) characterized by the presence of autoantibodies and multiorgan involvement. Various autoantibodies have been recognized in these patients, primarily targeting double-stranded DNA, Ro/La antigens and membrane phospholipids in SLE, pSjD and APS [1–3]. Notably, SRD are more prevalent in women of reproductive age, and they lead to a higher risk of adverse pregnancy outcomes (APO) such as pregnancy loss, preeclampsia and fetal growth restriction (FGR) [4].

Despite known clinical risk factors for APO development, the pathogenesis underlying APO is not fully understood in patients with SRD [5]. The placenta develops during pregnancy and plays a crucial role in maternal-fetal interactions necessary for fetal growth. After trophoblast invasion, subsequent vascular remodeling and diverse maternal immune adaptation processes are followed to tolerate the semi-allogenic fetus and achieve a successful pregnancy [6]. Decidual natural killer (dNK) cells and decidual macrophages constitute the predominant leukocytes populations and have a pivotal role in regulating trophoblast invasion, tissue reconstruction, angiogenesis and uterine arteries remodeling which are essential processes for successful placentation [7–9]. Besides, dysfunction of these cells during decidualization has been linked to recurrent pregnancy loss and APO such as preeclampsia and FGR [10–12].

In recent years, placental lesions have been proposed as a cause of APO in patients with SLE and/or APS [13], with uterine vascularity abnormalities and coagulopathy as the main findings [14]. Chronic inflammation, decidual vasculitis and complement deposition have also been described in placentas of SRD patients [15]. Moreover, increased perivillous fibrin deposition has been recently associated with small for gestational age offspring in SLE patients [16].

Presence of antiphospholipid antibodies (aPL) in SLE and APS patients has been linked with placental infarcts, ischemic villous lesions, placental insufficiency and subsequent APO [13, 17]. However, these autoantibodies do not fully explain the increased risk for APO in these diseases [18]. Unfortunately, data assessing placental manifestations in SRD is scarce and there are no studies that explore placental lesions in depth, for example concerning interferon (IFN) or changes in immune cells count, and their association with APO in different groups of SRD patients.

Type I IFN pathway activation has been related to SRD pathophysiology [19–22]. During pregnancy, SLE patients with preeclampsia had higher IFNα activity before clinical symptoms compared to those with an adverse outcome different from preeclampsia and healthy controls. Remarkably, preeclampsia itself was not associated with high IFNα activity in women without autoimmune disease [23]. IFNα expression was previously detected in intervillous blood samples from placentas of SLE patients. Nevertheless, due to the sample size, no association with placental lesions or APO was described [24]. In contrast, type II IFNγ is linked with preeclampsia development through the inhibition of extravillous trophoblast cell invasion via apoptosis and reduction of metalloproteinases in women without SRD [25].

Type I and II IFN-inducible gene families are upregulated in patients with APS compared to healthy controls (HC) and seem to differ from IFN-upregulation in SLE patients [26, 27]. Interestingly, a high prevalence of IFN-I gene signature in peripheral blood was positively correlated with preeclampsia history in a cohort of thrombotic APS patients without SLE-related anti-dsDNA or anti-Smith antibodies [28]. In pSjD patients, IFN type I pathway activation has been confirmed but to a lower degree than in SLE patients and without a clear association with disease activity [21]. Nevertheless, there is a lack of evidence concerning the IFN type I upregulation in placental tissue of patients with SRD.

Although placental damage is likely influenced by pathophysiological processes related to SRD and may contribute to APO development, the extent to which immune cell imbalances and IFN pathway activation play a role remains unclear. Furthermore, it is not well understood whether these immune alterations are solely attributable to SRD or also occur in women with APO in the absence of these diseases. Altogether, these SRD may share a common pathological pathway to develop APO and similar placental immune imbalances. In this study, we aimed to identify immunological imbalances in placentas of patients with SLE, pSjD or APS, focusing on IFN and cell subsets compared to healthy pregnancies and those with APO but without SRD.

## Methods

All available placenta biopsies from pregnant women treated at the University Medical Center Groningen (UMCG) between 2008 and 2022 who fulfilled the classification criteria for SLE (≥ 4 American College of Rheumatology (ACR) criteria or ≥ 4 Systemic Lupus Erythematosus International Collaborating Clinics (SLICC) criteria) [1, 29], pSjD (≥ 4 points in ACR/European League Against Rheumatism (EULAR) criteria) [2] or APS [30] were recovered from the pathology biobank. Women with SRD treated at the UMCG during the specified period were excluded from the study if the placenta was not collected or if the stored tissue was insufficient for analysis. In addition, healthy pregnancies from the NORMA cohort were included [10]. The NORMA cohort comprised pregnancies with a neonatal birth weight between p10-p90, blood pressure within normal range and without complications or medication use. Women with APO but without SRD were included. These positive APO controls comprised three groups: spontaneous preterm birth without preeclampsia (PTB), FGR without preeclampsia, and FGR with preeclampsia (FGR/PE). Healthy and positive APO controls were attended between 2008 and 2022 at the Obstetrics and Gynecology department of our hospital.

Only one pregnancy per patient was included and demographic data were obtained from medical records. Disease activity was assessed with the Systemic Lupus Erythematosus Disease Activity Index (SLEDAI) or EULAR Sjögren’s syndrome disease activity index (ESSDAI) [31, 32]. Preconception comorbidities such as arterial hypertension, chronic kidney disease or any previous thromboembolic events were recorded. Furthermore, use of assisted reproductive technology (ART), smoking habits, obstetric history and medication use during pregnancy were documented. This study was conducted in accordance with the Declaration of Helsinki, the Dutch law on Medical Research in Humans and approved by the Institutional Review Board (or Ethics Committee) of the UMCG (METc 2022/427 on the 30th of August 2022). Processing placental tissue was approved by the aforementioned and no informed consent was required according to our regulations and no informed consent was required according to our regulations.

### Adverse pregnancy outcomes definition

We limited our outcomes of interest to the presence of preterm birth (before the 37th week of gestation), severe preterm birth (before the 34th week of gestation), FGR [33], preeclampsia/eclampsia [34], and thromboembolic events during pregnancy or postpartum. A compound APO outcome was defined as the presence of at least one of the aforementioned.

### Immunohistochemistry

After delivery, placentas were fixed in formalin and one full-thickness sample of parenchyma from within the central two-thirds of the placental disc was taken and embedded in paraffin. Samples were taken according to the Amsterdam Placental Workshop Group recommendations [35]. Immunohistochemistry (IHC) was performed using the Ventana Benchmark Ultra machine (Ventana, USA) according to the manufacturer indications for Myxovirus resistance protein 1 (MxA), CD3, CD20, CD56, CD68 and CD123 or manually for Foxp3. Used primary antibodies and their dilution are described in Supplementary Table S1. For the manual IHC, slides were deparaffinized with xylene. Antigen retrieval was performed with 10 mM tris-HCL + 1 mM EDTA pH 9.0 in a water bath at 90 °C. Endogenous peroxidase was blocked using 0.3% H_2_O_2_ solution for 30 min. Subsequently, slides were washed with PBS three times for five minutes and incubated overnight at 4 °C with the primary antibody in PBS with 1% BSA. Slides were rewashed with PBS three times for five minutes, following incubation with secondary antibody in PBS with 1% BSA for one hour at room temperature. After washing with PBS three times for five minutes, slides were incubated with DAB (Dako, K4006) for 10 min. Hematoxylin counterstaining was performed, and subsequently, slides were dehydrated and covered with mounting medium and a cover slip for further analysis. In the Ventana machine, deparaffinizing and endogenous peroxidase blocking was performed automatically at 72 °C.

### Immune cell analysis

Slides were scanned with the Philips Intellisite Pathology Solution Ultra-Fast scanner 1.6.1.1.12. Digital analysis was performed using Visiopharm version 2023.01. Due to the complexity of placenta tissue and the high prevalence of lesions in our samples, a fetoplacental pathologist blinded to the subgroups’ background selected the areas of interest for analysis, leading to a different total analyzed area per patient. An automated detection classifier was built based on an initial manual discrimination of cells. A separate classifier for each immune cell subset was performed, and following color optimization, it was applied to all patients, obtaining the cell count discriminating between decidua (maternal) and chorionic villi (parenchyma) areas. All positive stained cells were analyzed as immune cells, and the analyzed area was given in mm^2^ for the analysis. An example of the classifier developed to analyze cells can be found in Supplementary Fig. 1.

### MxA and placental lesions assessment

Decidua and villi MxA expression was assessed semi-quantitatively by two pathologists blinded to the subgroups’ background on an ordinal scale from zero to two, with zero indicating no expression, one indicating weak diffuse expression, and two indicating diffuse solid expression. Representative images of the MxA scoring system can be found in Supplementary Fig. 2*.* In case of discrepancies, a second review was conducted, and consensus was achieved. This only happened in the decidua scoring of one FGR patient related to placental immaturity. Placental lesion classification was performed according to the definitions of the Amsterdam Workshop Group by a fetoplacental pathologist [35].

### Statistics

Data from continuous variables are presented as median with interquartile range (p25-p75). Categorical variables are presented as numbers with percentages. Overall differences between multiple groups in immune cell count and MxA levels were assessed with the Kruskal-Wallis test, and if significant, post-hoc Dunn’s test for pairwise comparisons was performed, with p-values adjusted using the Benjamini-Hochberg (BH) correction for multiple comparisons. Statistical significance was set as a two-sided p value less than 0.05 after correction. Statistical analyses were performed using IBM SPSS V28.0.1.0, IBM. Graphics were made using Prism, V9.1.0, GraphPad.

## Results

### Demographics and adverse pregnancy outcomes

Our study included placentas from 11 SLE, 4 pSjD, 8 APS, 4 PTB, 8 FGR, 8 FGR/PE patients and 11 HC. Demographic and disease characteristics before pregnancy are summarized in Table 1. Most of our population was Caucasian with a low rate of ART use. Notably, SLE and pSjD patients had low disease activity before conception.

**Table 1:** Characteristics of healthy controls, patients with systemic rheumatic diseases, fetal growth restriction or fetal growth restriction and preeclampsia

	HC (*n* = 11)	SLE (*n* = 11)	pSjD (*n* = 4)	APS (*n* = 8)	PTB (*n* = 4)	FGR (*n* = 8)	FGR/PE (*n* = 8)
Age (years)^1^	31 (29–36)	30 (28–34)	30 (27–35)	30 (25–36)	30 (27–33)	32 (26–39)	28 (23–31)
BMI before pregnancy (Kg/m^2^)^1^	-	24.2 (21.7–25.3)	23.7 (-)	32.1 (23.8–42.6)	-	-	-
Missing^3^	-	1	1	3	-	-	-
Gravidity^1^	3 (1–4)	1 (1–3)	2 (1–6)	3 (2–5)	1 (1–3)	2 (1–3)	1 (1–2)
Parity^1^	1 (0–2)	0 (0–1)	1 (0–3)	1 (0–2)	0 (-)	1 (0–2)	0
Systolic blood pressure (mmHg)^1^	-	117 (109–120)	105 (-)	120 (111–130)	-	-	-
Missing^3^	-	1	1	1	-	-	-
Race^2^							
Caucasian	10 (91%)	10 (91%)	4 (100%	7 (88%)	4 (100%)	8 (100%)	8 (100%)
Black	0	0	0	0	0	0	0
Other	1 (9%)	1 (9%)	0	1 (13%)	0	0	0
ART use^2^	2 (18.2%)	0	0	0	0	1 (12.5%)	0
Disease duration (years)^1^	-	6 (2–8)	2.5 (0.3–12.3)	6 (1.8-8)	-	-	-
Disease activity index before pregnancy^1,4^	-	2 (0-2.5)	0	-	-	-	-
Missing^3^	-	2	1	-	-	-	-
Tobacco use during pregnancy^2^	2 (18.2%)	2 (18.2%)	1 (25%)	1 (12.5%)	0	2 (25%)	3 (37.5%)
Arterial hypertension^2^	0	1 (9.1%)	0	1 (12.5%)	0	0	0
Chronic kidney disease^2^	0	1 (9.1%)	0	0	0	0	0
Lupus nephritis history^2^	-	1 (9.1%)	0	0	-	-	-
Any thrombo- embolic event history^2^	0	1 (9.1%)	0	5 (62.5%)	0	0	0

Data are presented as ^1^ median with the p25-p75 between brackets ^2^ or as number with the percentages between brackets; ^3^ Missing data for this variable; ^4^ Disease activity was assessed with the SLEDAI or ESSDAI indexes for SLE and pSjD patients, respectively. Abbreviations. HC, healthy controls; SRD, systemic rheumatic diseases; SLE, systemic lupus erythematosus; pSjD, primary Sjögren’s disease; APS, antiphospholipid syndrome; BMI, body mass index; ART, assisted reproductive technology PTB, spontaneous preterm birth; FGR, fetal growth restriction; PE, preeclampsia

Patients with SRD had a high rate of APO (70%) in our study. APS patients had a shorter pregnancy duration (209 days, IQR 185–260) compared to SLE (258 days, IQR 245–283) and pSjD (265 days, IQR 227–283) patients. Median SLEDAI and ESSDAI during pregnancy were 2 and 6 for SLE and pSjD patients, respectively. Two SLE and two pSjD patients had flares during pregnancy, mainly due to cutaneous manifestations that required immunomodulatory medication. The most common APO in SLE patients was PTB (54.5%), followed by FGR (27.3%), while APS patients had high rates of PTB (75%) and PE (63%). No thromboembolic events during pregnancy were described.

Neonates of APS patients had low birth weight (1300 g, IQR 718–2388) compared to SLE (2875 g, IQR 1875–3285) and pSjD (2658 g, IQR 1953–3360) patients, consistent with placental weight and achieved gestational age. Finally, high rates of low-dose oral glucocorticoid use were identified in SLE patients (63.6%) with concomitant HCQ therapy (91%). All APS patients used aspirin during pregnancy, most in combination with LMWH (88%). Other APO rates and medication use during pregnancy are described in Table 2.

**Table 2:** Pregnancy characteristics of healthy controls, patients with systemic rheumatic diseases, fetal growth restriction or fetal growth restriction and preeclampsia

	HC (*n* = 11)	SLE (*n* = 11)	pSjD (*n* = 4)	APS (*n* = 8)	PTB (*n* = 4)	FGR (*n* = 8)	FGR/PE (*n* = 8)
Gestational age at delivery (days)^1^	281 (273–294)	258 (245–283)	265 (227–283)	209 (185–260)	210 (201–216)	233 (216–266)	231 (202–260)
Disease activity index during pregnancy^1^	-	2 (0–2)	6 (-)	-	-	-	-
Missing^3^	-	2	1	-	-	-	-
Disease flares during pregnancy^2^	-	2 (18.2%)	2 (50%)	-	-	-	-
Missing^3^	-	-	1	-	-	-	-
APO presentation^2^	0	7 (63.6%)	2 (50%)	7 (87.5%)	4 (100%)	8 (100%)	8 (100%)
PTB (< 37 weeks)^2^	0	6 (54.5%)	1 (25%)	6 (75%)	4 (100%)	5 (63%)	6 (75%)
Extreme PTB (< 34 weeks)^2^	0	1 (9.1%)	1 (25%)	5 (63%)	4 (100%)	4 (50%)	4 (50%)
FGR^2^	0	3 (27.3%)	1 (25%)	1 (12.5%)	0	8 (100%)	8 (100%)
PE^2^	0	2 (18.2%)	1 (25%)	5 (63%)	0	0	8 (100%)
Thromboembolic event during pregnancy^2^	0	0	0	0	0	0	0
Newborn sex^2^							
Boy	4 (36.4%)	6 (54.5%)	2 (50%)	5 (62.5%)	3 (75%)	5 (62.5%)	3 (37.5)
Girl	7 (63.6%)	5 (45.5%)	2 (50%)	3 (37.5%)	1 (25%)	3 (37.5%)	5 (62.5%)
Birth weight (grams)^1^	3420 (3200–3940)	2875 (1875–3285)	2658 (1953–3360)	1300 (718–2388)	1525 (1263–1906)	1725 (1147–2183)	1655 (866–2213)
Placental weight (grams)^1^	535 (432–682)	389 (282–500)	427 (369–494)	262 (209–362)	278 (260–344)	276 (154–304)	253 (175–339)
Medication use during pregnancy							
Oral glucocorticoids use^2^	0	7 (63.6%)	0	0	0	0	0
Oral glucocorticoids dose (mg/day)^1^	0	5 (0-7.5)	0	0	0	0	0
HCQ use^2^	0	10 (91%)	1 (25%)	0	0	0	0
LMWH use^2^	0	3 (27.3%)	0	7 (88%)	0	0	0
Aspirin use^2^	0	5 (46%)	2 (50%)	8 (100%)	0	1 (12.5%)	0
DMARD use^2^	0	3 (27.3%)	0	0	0	0	0

Data are presented as ^1^ median with the p25-p75 between brackets or as ^2^ number with the percentages between brackets; ^3^ Missing data for this variable. Abbreviations. HC, healthy controls; SRD, systemic rheumatic diseases; SLE, systemic lupus erythematosus; pSjD, primary Sjögren’s disease; APS, antiphospholipid syndrome; PTB, preterm birth; FGR, fetal growth restriction; PE, preeclampsia

### Immune cell imbalances in placenta from patients with systemic rheumatic diseases

**Fig. 1:**
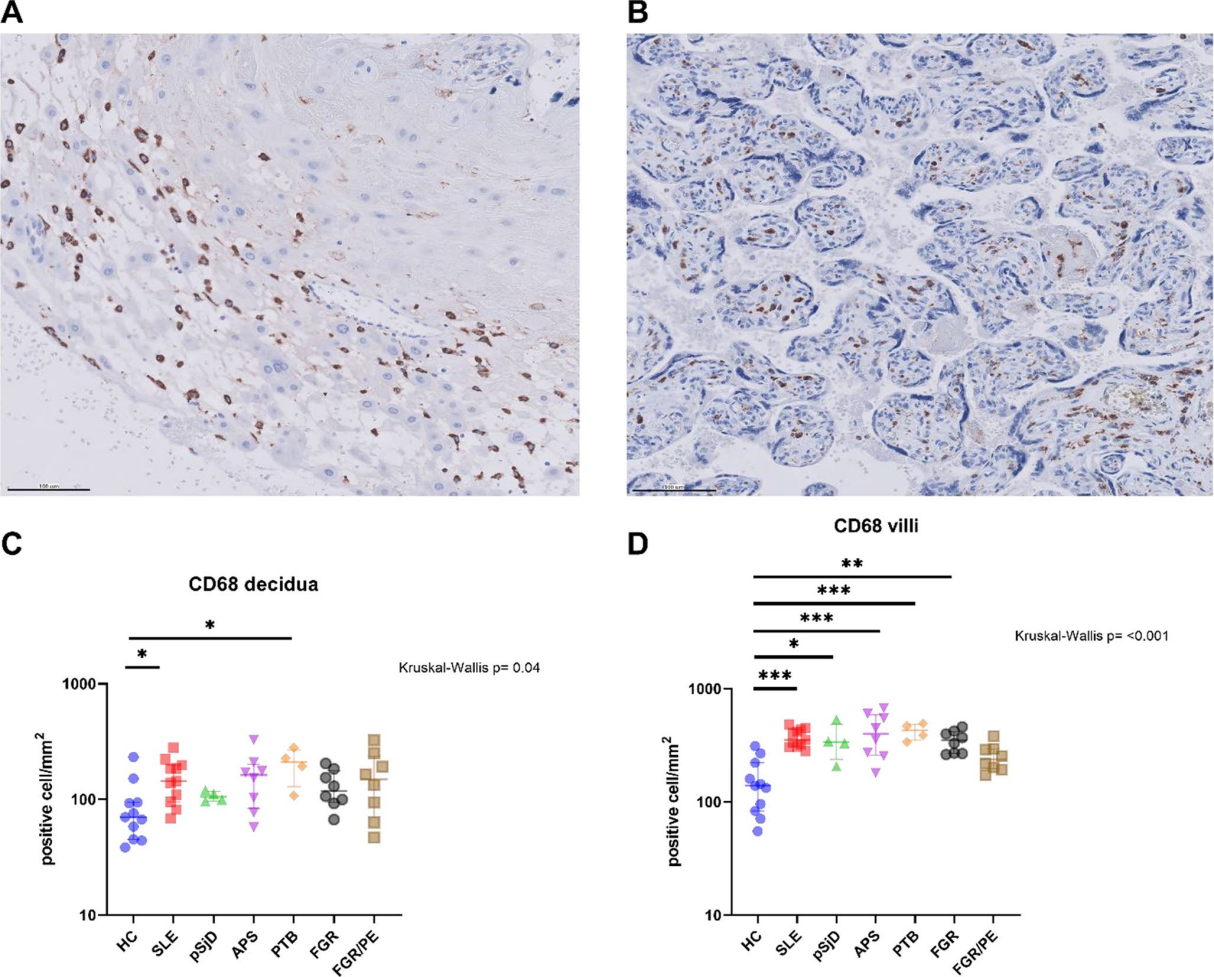
Macrophages in decidua and villi of healthy controls and patients with systemic rheumatic diseases, FGR and FGR/PE. Representative images of macrophages (CD68+) in (**A**) decidua and (**B**) villi of one SLE patient, together with quantification of cells per area in (**C**) decidua and (**D**) villi. Abbreviations. ns, no significant; *: p value < 0.05; **: p value ≤ 0.01; ***: p value ≤ 0.001

We analyzed the number of positive cells per square millimeter for the different stainings. Interestingly, patients with SRD had a higher macrophage (CD68+) count in decidua and villi than HC (Fig. 1, C-D). In decidua, this difference was found in the placentas of SLE patients, while in villi, pSjD and APS patients also had a higher macrophage count. Concerning patients without SRD, in decidua, only PTB shows an increased macrophage count compared to HC. In villi, this difference was significant for PTB, and FGR patients.

**Fig. 2:**
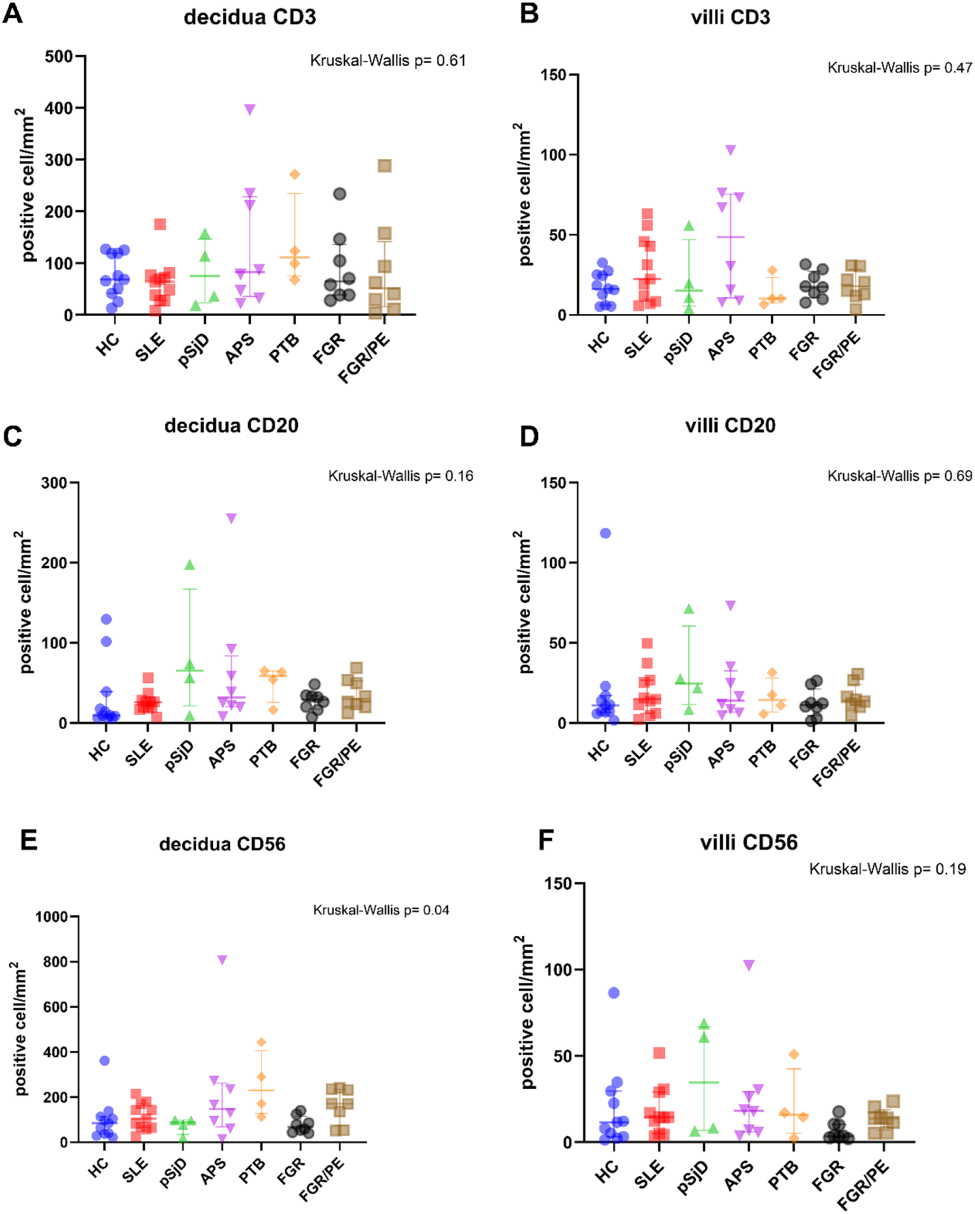
Placental lymphocyte count in (**A, C, E**) decidua and (**B, D, F**) villi of healthy controls and patients with systemic rheumatic diseases, FGR and FGR/PE. (**E**) No significant pairwise comparisons after Benjamini-Hochberg multiple testing correction. Abbreviations. ns, no significant; *: p value < 0.05; **: p value ≤ 0.01

Regarding other immune cells, no differences were observed in T (CD3+), B (CD20+), and NK (CD56+) lymphocyte count in decidua and villi between patients with SRD, PTB, FGR, FGR/PE and HC as shown in Fig. 2, A-F. Foxp3 T regulatory cells were identified in very low numbers in decidua and not in villi, and there were no differences between the groups. Next, we determined the Foxp3/CD3 ratio without finding significant differences between the groups (Supplementary Figure S3). Finally, no plasmacytoid dendritic cells (CD123+) were identified during analysis.

### MxA villi expression differs between patients with systemic rheumatic diseases and healthy controls

**Fig. 3:**
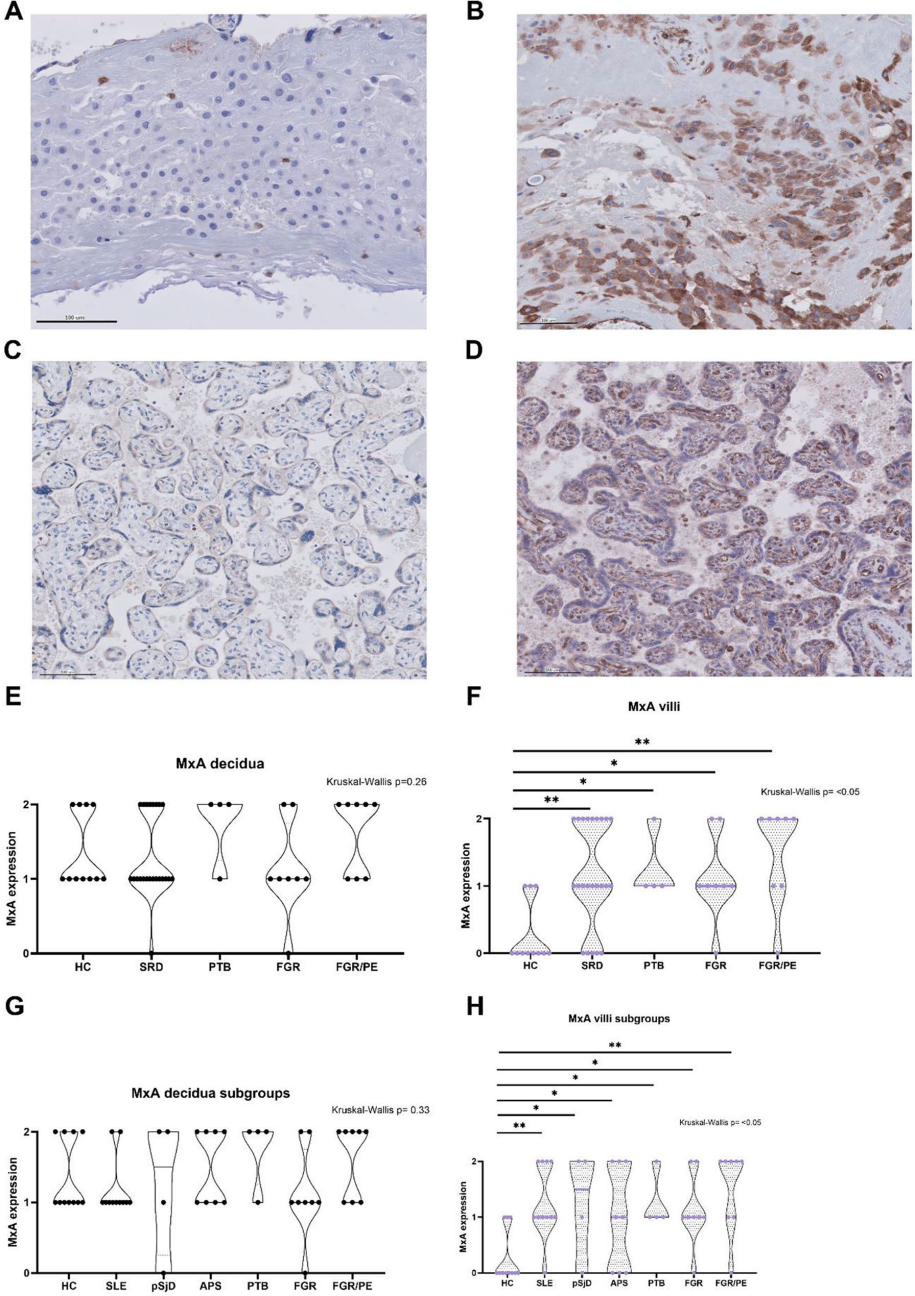
MxA expression in decidua and villi of healthy controls and patients with systemic rheumatic diseases, FGR and FGR/PE. Representative images of MxA expression scored as zero (no expression) in (**A**) decidua of one FGR and (**C**) villi of one HC and representative images of MxA expression scored as two (diffuse solid expression) in (**B**) decidua of one APS and (**D**) villi of one SLE patient. Furthermore, (**E, F**) quantification of MxA expression for combined SRD or (**G, H**) by subgroups. Abbreviations. ns, no significant; *: p value < 0.05; **: p value ≤ 0.01

Representative images of MxA expression with different scores (from zero to two in an ordinal scale) are depicted in Fig. 3, A-D. Patients with SRD had higher MxA values than HC in villi but not in decidua (Fig. 3, E-H). Furthermore, PTB, FGR and FGR/PE groups were associated with a higher MxA expression in villi compared to HC, which was not the case for decidual tissue (Fig. 3, E-H).

### High prevalence of placental lesions in SRD and non-rheumatic APO patients

Maternal vascular malperfusion (MVM), fetal vascular malperfusion (FVM), villitis of unknown etiology (VUE), chorioamnionitis (CA), chronic histiocytic intervillositis (CHIV), chorangiosis (CH) and increase in nucleated fetal red blood cells (FH) were identified in patients as depicted in Table 3. No patients with maternal floor infarction or massive perivillous fibrin deposition were identified. While SLE patients had a high prevalence of CA (55%), APS patients had higher rates of MVM (75%) and VUE (38%). On the other hand, PTB patients had high rates of CA (75%), while FGR and FGR/PE were characterized by MVM (38% and 88%, respectively). Placental lesions were frequent in our study, and all PTB, FGR, and FGR/PE had at least one type of placental lesion, while 18% of HC did not have any type of placental lesions.

**Table 3:** Distribution of placental lesions across patients

	HC (*n* = 11)	SLE (*n* = 11)	pSjD (*n* = 4)	APS (*n* = 8)	PTB (*n* = 4)	FGR (*n* = 8)	FGR/PE (*n* = 8)
MVM	0	1 (9.1%)	0	6 (75%)	0	3 (37.5%)	7 (87.5%)
FVM	3 (27.3%)	2 (18.2%)	0	2 (25%)	1 (25%)	2 (25%)	0
VUE	3 (27.3%)	2 (18.2%)	1 (25%)	3 (37.5%)	0	2 (25%)	2 (25%)
CA	3 (27.3%)	6 (54.5%)	1 (25%)	2 (25%)	3 (75%)	1 (12.5%)	1 (12.5%)
CHIV	0	0	0	1 (12.5%)	0	0	0
CH	5 (45.5%)	3 (27.3%)	1 (25%)	1 (12.5%)	0	2 (25%)	0
FH	0	1 (9.1%)	1 (25%)	0	0	2 (25%)	0
NL	2 (18.2%)	2 (18.2%)	1 (25%)	0	1 (25%)	0	0

Placental lesions were classified according to the definition of the Amsterdam Workshop Group. Data are presented as number with the percentages between brackets. Abbreviations. HC, healthy controls; SRD, systemic rheumatic diseases; SLE, systemic lupus erythematosus; pSjD, primary Sjögren’s disease; APS, antiphospholipid syndrome; PTB, spontaneous preterm birth; FGR, fetal growth restriction; PE, preeclampsia; MVM, maternal vascular malperfusion; FVM, fetal vascular malperfusion; VUE, villitis of unknown etiology; CA, chorioamnionitis; CHIV, chronic histiocytic intervillositis; CH, chorangiosis; FH, increase in nucleated fetal red blood cells; NL, no lesions

## Discussion

Our study determined that patients with SLE, pSjD and APS have a higher macrophage count and IFN upregulation in the placenta compared to HC. In concordance, higher proportions of macrophages have been described in decidua from obstetric APS patients compared to healthy women and those with unexplained recurrent spontaneous abortion without SRD [36]. Higher placenta macrophage count was also identified in other SRD, such as systemic sclerosis and undifferentiated connective tissue disease [37]. In our study, this increment seems differential between maternal and fetal tissue since decidua macrophage upregulation was present only in SLE and PTB patients, while it was present in all groups except FGR/PE in the villi. Contrary to previous reports, we did not find an increased decidual macrophage count in FGR or FGR/PE patients, probably due to our sample size and the use of different markers and/or different placenta sampling location [10, 38].

The period during pregnancy in which macrophage upregulation occurs, their phenotype switching and interactions with other immune cells in the maternal-fetal interphase in patients with SRD are poorly understood. In healthy women, decidual macrophages are crucial players in placenta development during pregnancy, having different functions in each trimester of pregnancy allowed by their phenotypic plasticity [39]. Decidual macrophages polarization is often divided into classically activated proinflammatory M1 and alternatively activated anti-inflammatory M2 macrophages. Remarkably, during the first stages of pregnancy, a proinflammatory phenotype is prevalent, allowing trophoblast invasion and survival during implantation, for later on, a switch to an M2 phenotype, which allows tissue growth, immunomodulation and homeostasis after the first trimester [39]. Interestingly, a proinflammatory M1-like macrophage phenotype has been described in the placentas of patients with PE, and a lower CD206+/CD68 + ratio was reported in FGR compared to HC [40]. Due to their role in SRD itself, it would be very interesting to investigate this further in those patients. Recently, new clusters of decidual macrophages have been proposed through single cell transcriptomics. Patients with obstetric APS had more HLA-DR^high^ and less CD11c^high^ decidual macrophages than healthy women, highlighting a proinflammatory local environment. While HLA-DR^high^ macrophages are involved in antigen presentation, phagocytosis and complement activation, CD11c^high^ macrophages promote angiogenesis and growth [36].

IFN signature is a key marker in the pathogenesis of several SRD, and it has been associated with PE presentation in SLE and primary APS patients, however, there is no data concerning IFN immunohistochemical expression in the placenta of patients with SRD [23, 28, 41]. IFN-γ (type II) overexpression was identified in trophoblast and vascular endothelium in the placenta of patients with PE compared with HC [42]. Our results indicate that in patients with SRD IFN upregulation is not only present in the periphery but also in placental tissue, especially on the fetal side. Nevertheless, the question arises as to which cells are responsible for IFN release, whether they are derived from maternal cells in the maternal-fetal interphase or if it is a fetal cell response after exposure to proinflammatory molecules from maternal blood. A recent study by Ding, et al. described radical S-adenosyl methionine domain containing 2 (RSAD2) as a pathogenic interferon-stimulated gene associated with inflammatory lipid accumulation in decidua and chorionic villi of SLE patients. Notably, this study identified macrophages as the most responsive decidua-derived immune cells to IFNα stimulation, suggesting their potential involvement in placental damage mediated by this pathway [43].

Although patients with APO but without SRD also had high IFN expression in villi compared to HC, determining whether IFN upregulation is the etiology or consequence of placental damage in SRD requires comparing patients who did and did not develop APO. Unfortunately, our study could not determine this due to its small sample size, retrospective design, and the high prevalence of APO in our cohort; thus, we encourage future studies to address this question.

Therapeutic options targeting interferon could be useful during pregnancy in patients with SRD. Indeed, HCQ treatment has been associated with better pregnancy outcomes in SLE and refractory APS patients due to its wide variety of immunomodulatory effects, including IFN regulation [44]. Besides, novel therapeutics with a direct effect on the IFN pathway such as anifrolumab could be valuable in patients with SRD during pregnancy. However, the impact of IFN-targeted therapy on placental development has not been studied, and further research on safety and efficacy should be carried out.

Changes in lymphocyte populations during pregnancy have been identified in patients with SLE. For instance, lower CD4 + T cell count in peripheral blood was associated with disease activity and IFNα positivity during pregnancy [45]. Moreover, decreased T regulatory cells have been linked to history of spontaneous abortion in these patients [46]. Despite the well-known participation of T and B cells in the pathophysiology of SRD, we did not find differences in the cell count between patients and HC in the placenta. On the contrary, increased numbers of T cells were recently described in decidua from obstetric APS patients by immunofluorescence [36]. Although we acknowledge our small sample size, our findings could also support the idea that placenta dysfunction is led by innate immune components rather than adaptive immunity responses as has been described in PE pathogenesis [47]. Furthermore, we described very small populations of Foxp3 + T regulatory cells only in the maternal side of the placenta in accordance with previous reports [10].

On the other hand, although NK cells have been described as the most abundant leukocytes during pregnancy, patients with SRD did not show a concomitant increase in decidual macrophages and NK cells. Premature NK cell activation has been associated with tolerance breakdown at the maternal-fetal interphase and subsequent PTB presentation [12]. Thus, NK subtype analysis and functional assays concerning these lymphocytes should be further explored at different time points during pregnancy in patients with SRD.

Compared to previous studies, a small proportion of HC did not have any type of placental lesions. However, this was mainly explained by the inclusion of CH in our classification and its high prevalence in this group [10]. As expected, placental lesions differ between SRD patients, being APS characterized by MVM and VUE while SLE had high rates of CA which correlates with the different APO in each group.

Although most SLE and pSjD patients had a low disease activity, no systemic flares, and active treatment during pregnancy, we observed a high prevalence of APO, in accordance with previous clinical studies in our hospital [5]. Notably, we recognized this high APO rate because of a possible selection bias, as pathology assessment of placentas from SRD patients without APO was not a routine practice at our hospital. This limitation is shared with other studies in this topic, which highlight the need for prospective collection of placentas from patients with SRD regardless of pregnancy outcome [16].

No thromboembolic events during pregnancy or placental lesions such as maternal floor infarction or massive perivillous fibrin deposition were identified in APS patients, almost all of whom were on anticoagulant therapy. Hence, in contrast to the thrombotic origin of APO in aPL-positive patients, we suggest that a proinflammatory placental environment could play a principal role in placental development failures and placental damage that leads to organ insufficiency, as previously suggested for obstetric APS [36, 48].

We acknowledge the limitations of our study, being the most influential the retrospective design and the relatively small sample size in specific subgroups. As a result, unfortunately we could not compare immune cell counts between SRD patients with and without APO and lacked sufficient statistical power to develop models identifying independent variables associated with placental damage and APO occurrence in SRD patients. Moreover, due to our technique, we were unable to perform double stainings to co-localize specific markers of interest, such as Foxp3 T regulatory cells. We encourage the development of multicenter prospective studies with clinical follow-up during pregnancy and placental immune cell analysis to identify further relationships between immune changes during pregnancy, placental immune dysregulation and APO occurrence in SRD patients.

## Conclusion

SLE, pSjD and APS patients have an increased macrophage count and interferon upregulation in the placenta compared to HC. Similar findings were described in the chorionic villi of women with APO but without SRD. In our study, this upregulation seems to occur on the fetal side (chorionic villi) rather than the maternal side (decidua). Therefore, a pro-inflammatory environment might be key inducing placental dysfunction, which may lead to subsequent APO development.

## Electronic supplementary material

Below is the link to the electronic supplementary material.

Supplementary Material 1

Supplementary Material 2

## Data Availability

The data that support the findings of this study are displayed in the article and in the supplementary material. Others are available on request from the corresponding author due to ethics regulations.

## Declarations

### Note on congress abstracts and publication

A preliminary version of this manuscript’s abstract was presented at the American College of Rheumatology Convergence 2024 in Washington, DC and published in the corresponding conference proceedings with reference Arthritis Rheumatol. 2024; 76 (suppl 9). DOI: 10.1002/art.42992.

### Disclaimer

This manuscript has not been previously archived. No part of this manuscript, including text and graphics is copied or published elsewhere in whole or in part. Generative AI and AI-assisted technologies were not used in the preparation of this work.
