# Supplementary material for: Immunohistochemical analysis reveals higher Myxovirus resistance protein 1 expression and increased macrophage count in placentas from patients with systemic rheumatic diseases
Source: Rheumatol Int. 2025 Apr 4;45(4):90. doi: 10.1007/s00296-025-05856-w (PMC11971135; doi:10.1007/s00296-025-05856-w)
Supplement: Supplementary file 1 — Supplementary Material 1 [file 296_2025_5856_MOESM1_ESM.docx]

# Supplementary material

## Table S1. Antibodies used for manual and automated immunohistochemistry.

| **Antibody** | **Antigen retrieval** | **Code** | **Host** | **Dilution** | **Secondary antibody** | **Dilution** |
| --- | --- | --- | --- | --- | --- | --- |
| MxA | Ultra CC1 52m | R&D Syst MxA/Mx1 | Goat | 1:100 | Ultraview DAB kit | Undiluted |
| CD3 | Ultra CC1 36m | Ventana 2GV6 | Rabbit mono | Ready to use | Ultraview DAB kit | Undiluted |
| CD20 | Ultra CC1 36m | Ventana L-26 | Mouse | Ready to use | Ultraview DAB kit | Undiluted |
| CD56 | Ultra CC1 36m | Ventana MRQ-42 | Rabbit mono | Ready to use | Ultraview DAB kit | Undiluted |
| CD68 | Ultra CC1 52m | Dako PG-M1 | Mouse | 1:100 | Ultraview DAB kit | Undiluted |
| CD123 | Ultra CC1 64m | BD9F5 | Mouse | 1:100 | Ultraview DAB kit | Undiluted |
| Foxp3 | pH 9 | ABCAM Ab20034 (236A/E7) | Mouse | 1:100 | Dako K4001 | Undiluted |

**A B**


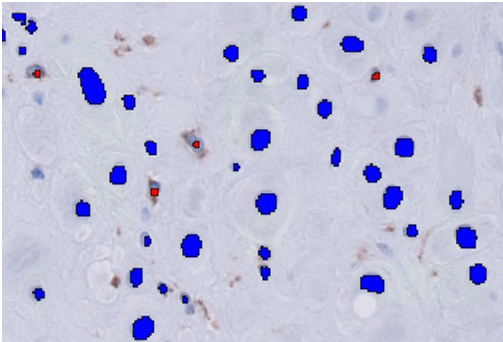

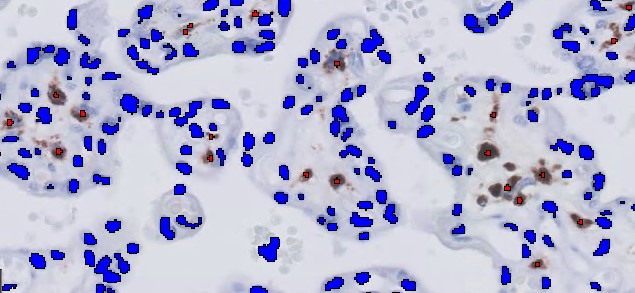


# **Fig. S1 CD68+ cell detection classifier.** Positive immune cells are pointed in red according to color DAB intensity. Negative cells corresponding to stromal and trophoblast cells are pointed in blue. The same strategy was performed for each staining after software independent training.


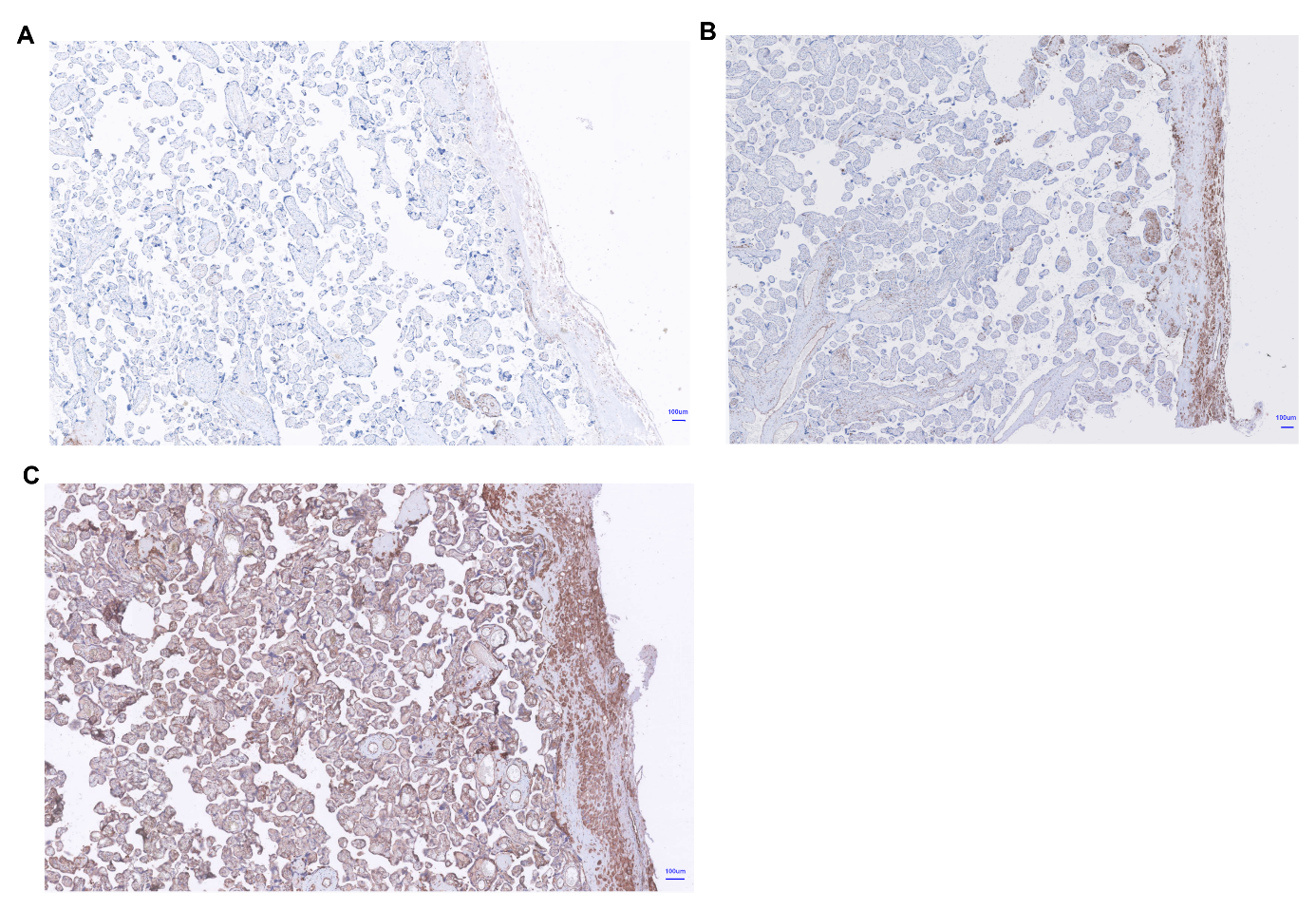


Fig. S2 MxA expression scoring system in decidua and villi. Representative images of MxA expression in decidua and villi scored as **(A)** zero (no expression) of a pSjD patient, **(B)** one (weak diffuse expression) of a SLE patient and **(C)** two (diffuse solid expression) of a SLE patient.

**
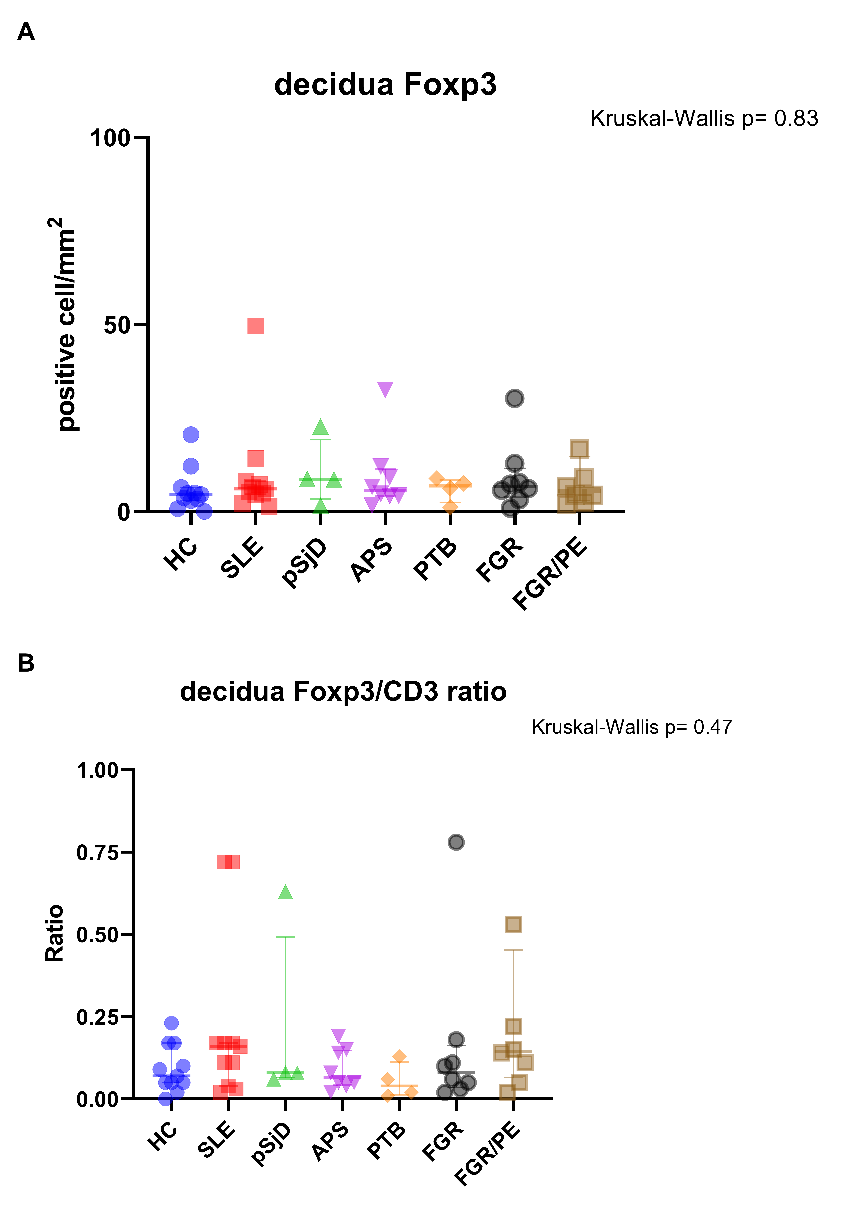
**

Fig. S3 Foxp3+ regulatory T cell count **(A)** and Foxp3/CD3 ratio **(B)** in decidua of healthy controls and patients with systemic rheumatic diseases, FGR and FGR/PE
